# Diagnostic performance of PET/CT in the detection of liver metastases in well-differentiated NETs

**DOI:** 10.1186/s40644-023-00556-9

**Published:** 2023-04-25

**Authors:** Freba Grawe, Natalie Rosenberger, Maria Ingenerf, Leonie Beyer, Ralf Eschbach, Andrei Todica, Ricarda Seidensticker, Christine Schmid-Tannwald, Clemens C. Cyran, Jens Ricke, Peter Bartenstein, Christoph. J. Auernhammer, Johannes Ruebenthaler, Matthias P. Fabritius

**Affiliations:** 1 Department of Radiology, University Hospital, LMU Munich, Marchioninistr. 15, 81377 Munich, Germany; 2 Department of Nuclear Medicine, University Hospital, LMU Munich, 81377 Munich, Germany; 3 Department of Internal Medicine 4, University Hospital, LMU Munich, 81377 Munich, Germany; 4 Interdisciplinary Center of Neuroendocrine Tumors of the GastroEnteroPancreatic System (GEPNET-KUM, ENETS certified Center of Excellence), University Hospital, LMU Munich, 81377 Munich, Germany

**Keywords:** NET, SSR, PET/CT, MRI, Liver metastases

## Abstract

**Background:**

The aim of this retrospective study was to compare the diagnostic accuracy of somatostatin receptor (SSR)-PET/CT to liver MRI as reference standard in the evaluation of hepatic involvement in neuroendocrine tumors (NET).

**Methods:**

An institutional database was screened for “SSR” imaging studies between 2006 and 2021. 1000 NET Patients (grade 1/2) with 2383 SSR-PET/CT studies and matching liver MRI in an interval of +3 months were identified. Medical reports of SSR-PET/CT and MRI were retrospectively evaluated regarding hepatic involvement and either confirmed by both or observed in MRI but not in SSR-PET/CT (false-negative) or in SSR-PET but not in MRI (false-positive).

**Results:**

Metastatic hepatic involvement was reported in 1650 (69.2%) of the total 2383 SSR-PET/CT imaging studies, whereas MRI detected hepatic involvement in 1685 (70.7%) cases. There were 51 (2.1%) false-negative and 16 (0.7%) false-positive cases. In case of discrepant reports, MRI and PET/CT were reviewed side by side for consensus reading. SSR-PET/CT demonstrated a sensitivity of 97.0% (95%CI: 96.0%, 97.7%), a specificity of 97.7% (95%CI: 96.3%, 98.7%), a PPV of 99.0% (95%CI: 98.4%, 99.4%) and NPV of 93.0% (95%CI: 91.0, 94.8%) in identifying hepatic involvement. The most frequent reason for false-negative results was the small size of lesions with the majority < 0.6 cm.

**Conclusion:**

This study confirms the high diagnostic accuracy of SSR-PET/CT in the detection of hepatic involvement in NET patients based on a patient-based analysis of metastatic hepatic involvement with a high sensitivity and specificity using liver MRI imaging as reference standard. However, one should be aware of possible pitfalls when a single imaging method is used in evaluating neuroendocrine liver metastases in patients.

**Supplementary Information:**

The online version contains supplementary material available at 10.1186/s40644-023-00556-9.

## Background

Neuroendocrine tumors (NETs) are relatively rare malignancies and thought to arise from cells throughout the diffuse endocrine system [1, 2]. Well-differentiated NETs are characterized by an indolent clinical course and relatively good prognosis [3, 4]. Nevertheless, patients at first diagnosis often present with locally advanced disease or distant metastases, with the liver as the most common site of metastatic disease [5, 6]. Neuroendocrine liver metastases (NELMs) cause significant impairment in quality of life and are one of the most important factors for long-term survival of NET patients [1, 7–10]. Therefore, the optimal management of the NELM is of utmost importance and accurate imaging is essential for assessing resectability, determining liver-targeted therapies, and monitoring remission. According to the World Health Organization (WHO), tumor classification of NETs is based on the proliferation index (KI-67) in grade 1, 2 and 3 [11]. Grade 1 and low Grade 2 NETs have high somatostatin receptor (SSR) expression, specifically the SSR subtype 2, in their cell membrane (80–95%), whereas grade 3 NETs show higher glucose metabolism and less SSR-expression [7, 12]. Thus, a high sensitivity of SSR-based PET/CT has been reported in G1 and G2 NETs with lower KI-67, while in G2 NETs with higher KI-67 and in G3 NETs the sensitivity of SSR-based PET/CT is substantially decreasing [13–15]. For diagnostic and therapeutic purposes, the affinity of radiolabelled somatostatin-analogues (SSR-analogues: e.g., 68Ga-DOTATATE, 68Ga-DOTATOC) to SSR forms the basis for the use of SSR-PET/CT (positron emission tomography/computed tomography) [15–17]. While conventional cross-sectional imaging represented the method of choice in NET patients for many years, SSR-PET/CT is increasingly used in staging, preoperative imaging and restaging and is recommended by many expert groups such as the National Comprehensive Cancer Network (NCCN), the European Neuroendocrine Tumor Society (ENETS), the European Association of Nuclear Medicine (EANM),the European Society of Medical Oncology (ESMO) and various national guidelines [5, 18–23]. Moreover, sufficient SSR-expression makes the patient eligible for peptide receptor radionuclide therapy [23, 24]. However, in the detection of NELMs MRI of the liver represents a well-established, widely used method and is particularly preferred in initial staging and preoperative diagnostic workup. Multiparametric MRI with i.v. hepatocyte-specific contrast agent, especially with repeated acquisitions using multiple sequences (dynamic contrast-enhanced MRI), including diffusion-weighted imaging (DWI) is considered the first choice for imaging to evaluate hepatic involvement and is particularly advantageous in young patients as there is no radiation exposure [25, 26]. However, there is a group of patients who cannot receive MRI to evaluate the liver due to contraindications (e.g. pacemakers, metal fragments) or claustrophobia. The aim of this study was to compare the diagnostic accuracy of SSR-PET/CT to multiparametric liver MRI as reference standard in the evaluation of hepatic involvement in neuroendocrine tumors.

## Methods

This study, which was conducted according to the Helsinki Declaration of 2013, was approved by the Ethics Committee of the Medical Faculty of the Ludwig Maximilian University Munich. Informed patient consent was waived due to the retrospective nature of the study.

### Study design and patient cohort

Our institutional database was screened for the term “SSR” imaging studies between 2006 and 2021 and a total of 8077 results were found. These imaging studies were assigned to a total of 2605 patients. All studies listed multiple times to one patient, studies of patients < 18 years or of other entities than G1/G2 NETs of the aerodigestive tract (e.g., meningioma, paraganglioma) were excluded. Of the therefore remaining 1570 patients, exactly 1000 patients received at least one MRI of the liver within 3 months after a matching SSR-PET/CT study. Due to the possibility of analysing matching examinations of one patient from more than one time point, a total of 2383 SSR-PET/CT imaging studies and matching multiparametric MRIs of the liver were included in the final analysis. Multiparametric MRI imaging was regarded as the reference standard.

**Fig. 1:**
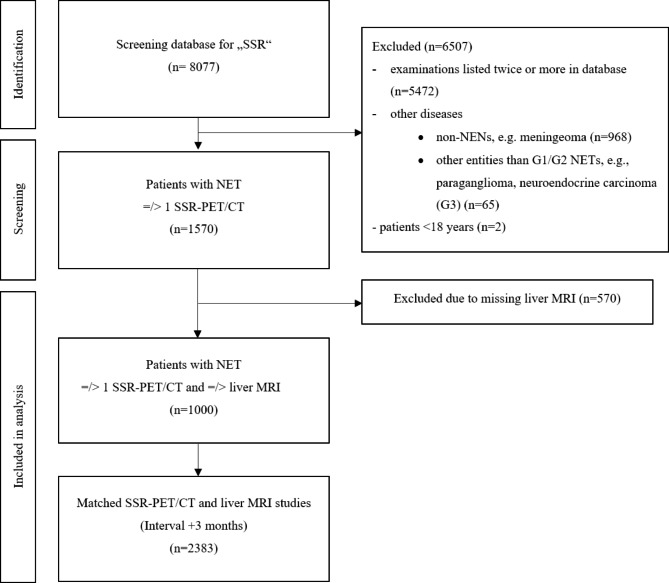
Flow chart of patient selection

We retrospectively investigated the medical reports of all SSR-PET/CT and multiparametric MRI examinations included in this study regarding patient-based metastatic hepatic involvement. Therefore, medical reports of SSR-PET/CT and MRI were retrospectively classified as hepatic involvement and non-hepatic involvement. Medical reports were created as follows in our clinical routine: SSR-PET/CTs were evaluated by four readers: one experienced and board-certified radiologist, one experienced and board-certified nuclear medicine physician, one radiology resident, and one nuclear medicine resident. MRI was analysed by an experienced, board-certified radiologist and a radiology resident. Hepatic-/non-hepatic involvement was either confirmed by both, SSR-PET/CT and MRI, or described in MRI but not in SSR-PET/CT (false-negative) or in SSR-PET/CT but not in MRI (false-positive). In case of divergent medical reports, additional imaging studies - and in unclear solitary lesions additional pathological reports - were reviewed side by side by an independent reader to find the underlying reason and reach final consensus. Patient selection is presented in Fig. 1.

### SSR-PET/CT imaging

SSR-PET/CT scans were acquired on a Discovery 64-slice PET/CT scanner (GE Healthcare) (n = 33) or a Biograph 64 TruePoint w/TrueV and Biograph mCT Flow 20-4R PET/CT scanner (Siemens, Healthcare GmbH, Erlangen, Germany) (n = 2350) and were initiated approximately 60 min after intravenous administration of a standard amount of approx. 180 MBq radiolabelled somatostatin analogues (68Ga-DOTATOC and 68Ga-DOTATATE). After intravenous injection of contrast agent 1.5 times the body weight (Ultravist 300, Bayer Vital GmbH, Leverkusen, Germany or Imeron 350 mg/mL, 2.5 ml/s, Bracco Imaging Deutschland GmbH, Konstanz, Germany) diagnostic venous-phase CT scans of the neck, thorax, abdomen, and pelvis (100–190 mAs; 120 kV) were acquired. Patients received diagnostic CT scan without contrast enhancement in case of known allergic reactions to iodinated contrast agent, renal impairment/failure or hyperthyreoidism. Imaging construction was automatically performed using built-in software. 3 mm-slice reconstructions were used for reading.

### MR imaging

Multiparametric MR examinations were mainly performed on a 1.5 (Magnetom Avanto, Magnetom Aera, Siemens Healthcare, Erlangen, Germany; Ingenia S, Philips Healthcare, Hamburg, Germany) and occasionally on a 3 T MR system (Verio, Skyra, Vida, Siemens Healthcare, Erlangen, Germany; Ingenia, Philips Healthcare, Hamburg, Germany) using a phased-array-coil for signal reception. The usual liver imaging protocol contained unenhanced T1w gradient-echo (GRE) sequences in- and out-of-phase, a single shot T2w sequence, a T1w 3D GRE sequence with fat suppression (fs) before and 20, 50, and 120 s after intravenous contrast injection (Gd-EOB-DTPA; Primovist, Eovist, Bayer Schering Pharma, Germany; 25 mol/kg body weight), a multishot T2w turbo spin echo sequence (fs), diffusion-weighted sequences with b-values of at least 50, and 800 s/mm2, and a T1w GRE (fs) and a T1w 3D GRE (fs) after 15 min delay (hepatobiliary phase). All sequences were acquired with parallel imaging with an acceleration factor of 2. ADC maps were calculated with all b-values.

### Statistical analysis

All continuous variables were expressed as mean and standard deviation (SD). For statistical analysis, diagnostic accuracy of SSR-PET/CT was tested using sensitivity, specificity, positive predictive value (PPV) and negative predictive value (NPV). Chi-squared test was used to compare the frequency of false-positive and false-negative findings. Additionally, exact 95% confidence intervals (CI) were calculated for all values.

## Results

Of the 1000 patients enrolled in this study, 469 were female (47%) and 531 were male (53%). The mean age at first PET/CT scan was 58.6 ± 13.1 years (range: 18 to 88 years). The most frequent location of primary NET was gastroenteropancreatic (GEPNET; n = 856, 86%), followed by the lung (n = 54, 5%), carcinoma of unknown origin (CUP; n = 51, 5%) and other (n = 39, 4%). The administered SST-analogs in the performed SSR-PET/CT scans were 68Ga-DOTATATE (65%) or 68Ga-DOTATOC (35%).

### Hepatic involvement

A total of 2383 studies were analysed. Hepatic involvement was confirmed by MRI in 1685 (71%) cases. SSR-PET/CT identified hepatic involvement in 1650 (69%) cases. An overview is shown in Table 1.

**Table 1:** Hepatic involvement detected by MRI and/or SSR-PET/CT

	MRI		
**SSR-PET/CT**	positive	negative	N_total_
positive	1634	16	1650
negative	51	682	733
N_total_	1685	698	2383

### Hepatic involvement detected by MRI without findings in SSR-PET/CT (false-negative)

**Fig. 2:**
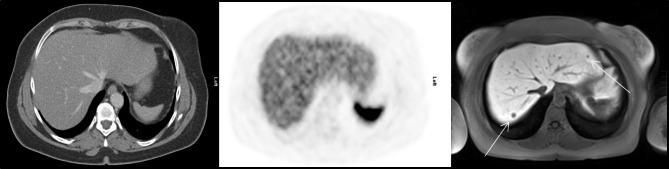
False-negative PET finding in a 52-year-old woman with a NET of the ileum. In the contrast-enhanced CT (left) no suspicious findings of the liver were detected; on PET (middle) no pathological SSR-uptake above the physiological SSR-uptake of liver parenchyma was detected. On hepatobiliary phase MRI (right), small liver metastases with overall low hepatic tumor burden were clearly delineated (arrows)

In 51 (2.1%) cases, hepatic involvement was reported in the MRI without corresponding findings in the SSR-PET/CT scan (false-negative). The most frequent reason for the lack of SSR-uptake in the SSR-PET/CT was the small size of lesions in patients with overall low hepatic involvement (n = 36), with mostly lesions < 0.6 (n = 30), and very few lesions with 0.7 cm (n = 3) and 1.2 cm (n = 3) measured in the MRI. In patients with low hepatic involvement and liver lesions located subcapsular, SSR-uptake could not be found in 6 cases. In 3 SSR-PET/CT scans, liver lesions were located close to the central hilar vessels and were rated falsely as vascular-associated SSR-uptake. In 2 SSR-PET/CT scans of patients with NET of the intestine, SSR-uptake of the liver metastases was low and rated as non-malignant. In one further scan, a hepatic metastasis with low SSR-uptake was stated as haemangioma. In 2 SSR-PET/CT scans, no SSR-uptake was reported but could be detected retrospectively correlating to the hepatic metastases detected by the MRI. In one patient with a 1.5 cm liver lesion, SSR-uptake could only be detected retrospectively due to high background noise of the liver. A summary of false-negative findings is presented in Table 2. An example for a false-negative finding in SSR-PET/CT is demonstrated in Fig. 2.

**Table 2:** False-negative findings in SSR-PET/CT

N	Finding in MRI	Finding in SSR-PET/CT
36	small lesions < 1.2 cm	no SSR-uptake
6	low hepatic involvement, lesions located subcapsular	no SSR-uptake
3	lesions located close to the big vessels	SSR-uptake rated as vascular-associated
2	low hepatic involvement with small lesions	low SSR-uptake rated as non-malignant
1	low hepatic involvement with small lesions	low SSR-uptake rated as haemangioma
2	low hepatic involvement with small lesions and insufficient co-registration of PET and CT	no SSR-uptake
1	high background noise of the liver	no SSR-uptake

### Hepatic findings in SSR-PET/CT without correlate in MRI (false-positive)

**Fig. 3:**
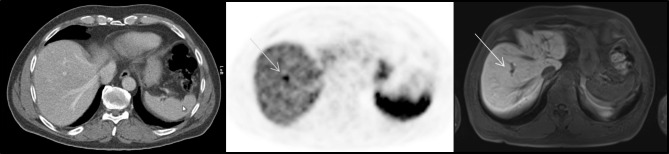
False-positive PET finding in a 65-year-old man with a NET of the caecum. In the contrast-enhanced CT (left) no suspicious findings of the liver were detected; on PET (middle) focal SSR-uptake above SSR-uptake of liver parenchyma was detected on hepatobiliary phase MRI (right), no suspicious liver lesions were found, most likely due to bile duct-associated SSR-uptake.

In 16 (0.7%) cases, SSR-PET/CT showed SSR-uptake in the liver without any correlating findings in the corresponding MRI (false-positive). Retrospectively, we analysed following reasons underlying the false positive findings: in 4 cases haemangiomas were identified in the MRI with low SSR-uptake in SSR-PET/CT. In another 4 cases liver cysts were detected in the MRI showing low, partly artificial SSR-uptake in SSR-PET/CT, partly due to insufficient co-registration. In 6 SSR-PET/CTs artificial SSR-uptake, e.g., vascular-associated SSR-uptake (Fig. 3), was found in the SSR-PET/CT without any corresponding findings in the MRI. In 2 cases extrahepatic physiological SSR-uptake (of stomach and small bowel) directly adjacent to the liver was falsely identified as hepatic uptake due to insufficient co-registration. False-positive findings are demonstrated in Table 3.

False-negative findings were statistically significantly more frequent than false-positive findings (p < 0.01).

**Table 3:** False-positive findings in SSR-PET/CT

N	Finding in MRI	Finding in SSR-PET/CT
4	haemangioma	low SSR-uptake
4	liver cysts	low, artificial SSR-uptake/ insufficient co-registration of PET and CT
6	no hepatic findings	artificial SSR-uptake (e.g. vascular-associated)
2	no hepatic findings	extrahepatic physiological SSR-uptake directly adjacent to liver

### Diagnostic accuracy of SSR-PET/CT

A total of 2383 SSR-PET/CT imaging studies were classified by the reference standard to calculate sensitivity and specificity. SSR-PET/CT reading showed a sensitivity of 97.0% (95%CI: 96.0%, 97.7%), a specificity of 97.7% (95%CI: 96.3%, 98.7%), a PPV of 99.0% (95%CI: 98.4%, 99.4%) and a NPV of 93.0% (95%CI: 91.0, 94.8%) for the detection of hepatic involvement in NET patients. Kappa for diagnostic accuracy was κ = 0.93 (95%CI: 0.92, 0.95).

## Discussion

Liver metastases in patients with NET are an indicator for poor prognosis and markedly reduced survival, which makes accurate staging of these patients crucial for best clinical management [7, 27–29]. Cross-sectional imaging, in particular multiparametric MRI imaging of the liver, represents the reference standard in the diagnosis, staging and restaging of NELMs [5]. SSR-PET/CT is recommended in all patients with grade 1 and grade 2 NET of the gastroenteropancreatic system and lung-NETs [19]. However, studies evaluating the diagnostic performance of SSR-PET/CT in the detection of NET hepatic involvement using multiparametric liver MRI as reference standard are lacking.

Our results demonstrate that SSR-PET/CT provides very high sensitivity (97.0%) and specificity (97.7%) in the detection of patient-based metastatic hepatic involvement in NET patients compared to the reference imaging modality multiparametric MRI with a high PPV (99.0%) and NPV (93.0%). Small series of NET patients have analyzed lesion-based metastatic hepatic involvement and reported a higher sensitivity of MRI imaging of the liver compared to SSR-PET/CT [30]. Armbruster et al. demonstrated higher diagnostic accuracy of 68Ga-DOTATATE-PET/CT parameters in distinction of NELMs and liver tissue compared to dynamic-contrast-enhanced MRI perfusion parameters; of note, highest accuracy was reached with the combination of both, as information of modalities is complementary [31]. Sadowski et al. examined the clinical utility of 68Ga-DOTATATE imaging for detecting unknown primary tumors and metastatic disease in a prospective study of 131 patients with suspected or known GEPNETs and compared it *inter alia* to CT/MRI. 68Ga-DOTATATE PET/CT had a detection rate of 95.2% compared with detection rates of 45.6% and 30.9% for anatomic imaging and patient management recommendations was altered in 32.8% of patients [32].

SSR-PET/CT imaging has a major impact on patients’ clinical management, as demonstrated in previous studies. Frilling et al. reported on the role of SSR-PET/CT in detection/staging of NET and altered treatment decisions in more than every second patient (31/52; 59.6%) compared to CT and/or MRI alone [33]. This was also the case in a study conducted by Hofman et al., who reported on a change in treatment plan in 47% of patients with NET after PET/CT imaging [34]. In concordance, management was changed in more than one third of patients undergoing SSR-PET/CT in a meta-analysis by Barrio et al. [35]. Furthermore, SSR-PET/CT offers a cost-effective imaging method modality that can be performed in one pass for detection of primary lesion and metastases and potentially save therapy costs due to early and time effective whole-body imaging [36, 37].

SSR-expression makes NET lesions not only targets for functional imaging but also selects patients for targeted therapy (peptide receptor radionuclide therapy, PRRT), which represents a systemic treatment option in inoperable, metastatic NET patients; the extent of SSR-expression in SSR-PET/CT indicates patients’ eligibility for treatment [38]. Large studies, such as the NETTER-1 trial demonstrated that PRRT lengthens the progression-free survival, time to health-related quality of life deterioration and increases survival probability [24, 39, 40].

However, since surgical treatment represents the only potentially curative treatment option in NET patients, accurate assessment of the extent of metastatic burden with regard to resectability has significant impact on clinical patient management, as well in primary tumor resection as in hepatobiliary surgery of NELMs [10, 27, 41].

Therefore, despite many advantages, some pitfalls and physiological processes regarding SSR-uptake in SSR-PET/CT must be considered. In our retrospective study analyzing patient-based metastatic hepatic involvement, the most frequent reason for negative SSR-PET/CT results was low spatial resolution of SSR-PET/CT compared to MRI in patients with low hepatic tumor burden and small liver lesions (< 1.2 cm). In patients with limited hepatic disease, it is known that MRI can be more sensitive for the detection of sub-centimeter liver metastases and should be performed in these cases in order to prevent hampering accurate staging of NET patients [34, 42, 43]. Moreover, MRI is free of ionizing radiation, which is especially advantageous in young patients [25, 26, 44]. Important to mention, according to ENETS consensus guidelines a hepatocyte-specific contrast-enhanced dynamic MRI should be performed including DWI, latter one known for its important role in oncological imaging offering a high lesion-to-background contrast in tumors and consequently increased diagnostic accuracy of especially small liver metastases [5, 45].

We observed that in patients with low hepatic involvement, beside the missing SSR-uptake, liver lesions were also not detectable in the contrast-enhanced CT of combined SSR-PET/CT images. This is in line with previous studies showing high sensitivity in NET only for multiphase CT (arterial, portal-venous and venous-phases), which is not standard clinical practice [46, 47].

Furthermore, it should be noted that unspecific, physiological SSR-uptake for example of the liver, small and large intestine and stomach might represent a potential source of false results in the detection of hepatic involvement in NET patients in the SSR-PET/CT [34]. Our study showed that in some patients, pathological SSR-uptake of liver metastases located close to organs with physiological SSR-uptake were falsely rated as non-malignant and vice versa extrahepatic physiological SSR-uptake (of stomach and small bowel) directly adjacent to the liver was falsely identified as pathological hepatic SSR-uptake. The same was observed for liver lesions close to the central hepatic vessels, where SSR-uptake was mistaken for vascular-associated uptake. Furthermore, in some cases exact co-registration of the combined PET and CT should be performed carefully to at least minimize the error rate in this regard. However, our results show that these cases occur very rarely.

False-negative findings were significantly more frequent than false-positive findings which emphasizes the high specificity of SSR-PET/CT [48]. SSR tracers show high affinity to somatostatin 2 and 5 receptors, which are overexpressed in NET. The higher false-negative rate is to be explained mainly due to the above-mentioned small sub-centimeter lesion size which was under the detection rate of the PET-study. by the above-mentioned rate of small sub-centimeter lesions.

We encountered only 3 patients with well-differentiated NET (Ki-67 2%) and low SSR-uptake of hepatic involvement. Higher-grade NETs and neuroendocrine carcinomas tend to have absent or low SSR-expression, leading to false-negative results. Although this was not the case in our study, the possibility of dedifferentiation in the clinical course of the disease must be considered in patients with low SSR-uptake (“flip-flop” phenomenon) and 18 F-FDG-PET/CT might be additionally performed for non-invasive histopathologic grading [49]. Furthermore, it is known that not all cells in the NET tumor mass show same differentiation levels resulting in not just intertumoral but also intratumoral heterogeneity resulting in variable SSR-uptake [13, 50]. For example, in insulinomas only relatively low sensitivity (64–67%) of 68Ga-PET/CT has been reported and was higher in malignant than in benign insulinomas [51, 52].

To our knowledge, this study represents the largest retrospective patient cohort evaluating patient-based metastatic hepatic involvement in SSR-PET/CT scans with validation in the reference imaging multiparametric MRI of the liver. There are a few limitations of this study. Firstly, this was a retrospectively conducted single-center study and histopathological confirmation was not given in all patients. In addition, expert reading was not blinded since medical reports were generated in clinical routine. SST-analogues (68Ga-DOTATATE, 68Ga-DOTA-TOC) varied with patients and examination. However, previous studies reported on no differences in diagnostic accuracy evaluating the sensitivities and uptake values among SSR-ligands, even regarding tumor origin or tumor grading [53–55]. The effective dose of both radiopharmaceuticals is comparable, which makes them even comparable from a radiation dosimetry point of view [56]. Furthermore, this retrospective chart analysis only compared patient-based sensitivity and not lesion-based sensitivity of SSR-PET/CT versus multiparametric MRI of the liver. While most NELMs were detected by SSR-PET/CT analyzing patient-based metastatic hepatic involvement, smaller studies based on a per lesion analysis have reported a higher detection rate of smaller NELMs by MRI. In a small study cohort of 32 NET patients, a lesion-based analysis of liver metastases was performed in 68Ga-DOTATATE PET and compared with CT or MRI. 68Ga-DOTATATE PET appeared to be at least equivalent or even superior to MRI or CT in the detection of NET liver metastases [30]. However, our data presented possible pitfalls and cases (e.g., small liver lesions, low tumor burden) in which MRI remains the essential diagnostic tool. The highest accuracy has been reported with the combination of multiparametric MRI liver and SSR-PET/CT as information of modalities is complementary [31]. Accordingly, a recent meta-analysis reported superior sensitivity of SSR-PET/MRI in comparison to SSR-PET/CT in the detection of NELMs [57]. Thus SSR-PET/CT and multiparametric MRI liver is recommended in patients with NETs, especially before evaluation for surgery.

## Conclusion

This study confirms the high diagnostic accuracy of SSR-PET/CT in the detection of hepatic involvement in NET patients based on a patient-based analysis of metastatic hepatic involvement with a high sensitivity and specificity using multiparametric liver MRI imaging as reference standard. SSR-PET/CT represents an important diagnostic tool and should be part of NET tumor staging and restaging. Moreover, it can serve as a stand-alone examination without high information loss in patients who cannot receive MRI. However, one should be aware of possible pitfalls when a single imaging method is used in evaluating neuroendocrine liver metastases in patients.

## Electronic supplementary material

Below is the link to the electronic supplementary material.

Supplementary Material 1

## Data Availability

The data that support the findings of this study are available from the corresponding author upon reasonable request.

## List of abbreviations

SSR: Somatostatin receptor
NET: In neuroendocrine tumors
NELMs: Neuroendocrine liver metastases
RE: Gradient-echo
PPV: Positive predictive value
NPV: Negative predictive value

## References

[1] Dasari A, Shen C, Halperin D, Zhao B, Zhou S, Xu Y, Shih T, Yao JC (2017). Trends in the incidence, prevalence, and survival outcomes in patients with neuroendocrine tumors in the United States. JAMA Oncol.

[2] Kulke M, Benson A, Bergsland E, Berlin J, Blaszkowsky L, Choti M, Clark O, Doherty G, Eason J, Emerson L (2012). Neuroendocrine tumors. J Natl Compr Canc Netw.

[3] Pavel M, Baudin E, Couvelard A, Krenning E, Öberg K, Steinmüller T, Anlauf M, Wiedenmann B, Salazar R (2012). ENETS Consensus Guidelines for the management of patients with liver and other distant metastases from neuroendocrine neoplasms of foregut, midgut, hindgut, and unknown primary. Neuroendocrinology.

[4] Yao JC, Hassan M, Phan A, Dagohoy C, Leary C, Mares JE, Abdalla EK, Fleming JB, Vauthey JN, Rashid A (2008). One hundred years after “carcinoid”: epidemiology of and prognostic factors for neuroendocrine tumors in 35,825 cases in the United States. J Clin oncology: official J Am Soc Clin Oncol.

[5] Sundin A, Arnold R, Baudin E, Cwikla JB, Eriksson B, Fanti S, Fazio N, Giammarile F, Hicks RJ, Kjaer A (2017). ENETS Consensus Guidelines for the Standards of Care in Neuroendocrine Tumors: Radiological, Nuclear Medicine & Hybrid Imaging. Neuroendocrinology.

[6] Schreiter NF, Nogami M, Steffen I, Pape UF, Hamm B, Brenner W, Röttgen R (2012). Evaluation of the potential of PET-MRI fusion for detection of liver metastases in patients with neuroendocrine tumours. Eur Radiol.

[7] Rinke A, Müller HH, Schade-Brittinger C, Klose KJ, Barth P, Wied M, Mayer C, Aminossadati B, Pape UF, Bläker M (2009). Placebo-controlled, double-blind, prospective, randomized study on the effect of octreotide LAR in the control of tumor growth in patients with metastatic neuroendocrine midgut tumors: a report from the PROMID Study Group. J Clin oncology: official J Am Soc Clin Oncol.

[8] Swiha MM, Sutherland DEK, Sistani G, Khatami A, Abazid RM, Mujoomdar A, Wiseman DP, Romsa JG, Reid RH, Laidley DT (2022). Survival predictors of (177)Lu-Dotatate peptide receptor radionuclide therapy (PRRT) in patients with progressive well-differentiated neuroendocrine tumors (NETS). J Cancer Res Clin Oncol.

[9] Panzuto F, Pusceddu S, Faggiano A, Rinzivillo M, Brighi N, Prinzi N, Riccardi F, Iannicelli E, Maggio I, Femia D (2019). Prognostic impact of tumour burden in stage IV neuroendocrine neoplasia: a comparison between pancreatic and gastrointestinal localizations. Pancreatology.

[10] Frilling A, Modlin IM, Kidd M, Russell C, Breitenstein S, Salem R, Kwekkeboom D, Lau WY, Klersy C, Vilgrain V (2014). Recommendations for management of patients with neuroendocrine liver metastases. Lancet Oncol.

[11] Nagtegaal ID, Odze RD, Klimstra D, Paradis V, Rugge M, Schirmacher P, Washington KM, Carneiro F, Cree IA (2020). The 2019 WHO classification of tumours of the digestive system. Histopathology.

[12] Rufini V, Baum RP, Castaldi P, Treglia G, De Gaetano AM, Carreras C, Kaemmerer D, Hommann M, Hörsch D, Bonomo L (2012). Role of PET/CT in the functional imaging of endocrine pancreatic tumors. Abdom Imaging.

[13] Liu X, Li N, Jiang T, Xu H, Ran Q, Shu Z, Wu J, Li Y, Zhou S, Zhang B (2020). Comparison of gallium-68 somatostatin receptor and (18)F-fluorodeoxyglucose positron emission tomography in the diagnosis of neuroendocrine tumours: a systematic review and meta-analysis. Hell J Nucl Med.

[14] Majala S, Vesterinen T, Seppänen H, Mustonen H, Sundström J, Schalin-Jäntti C, Gullichsen R, Schildt J, Kemppainen J, Arola J et al. Correlation of Somatostatin Receptor 1–5 Expression, [(68)Ga]Ga-DOTANOC, [(18)F]F-FDG PET/CT and Clinical Outcome in a Prospective Cohort of Pancreatic Neuroendocrine Neoplasms. Cancers 2021, 14(1). 10.3390/cancers14010162 PMC8750461 35008325

[15] Ambrosini V, Zanoni L, Filice A, Lamberti G, Argalia G, Fortunati E, Campana D, Versari A, Fanti S. Radiolabeled Somatostatin Analogues for Diagnosis and Treatment of Neuroendocrine Tumors. Cancers 2022, 14(4). 10.3390/cancers14041055 PMC8870358 35205805

[16] Beyer L, Gosewisch A, Lindner S, Völter F, Mittlmeier LM, Tiling R, Brendel M, Cyran CC, Unterrainer M, Rübenthaler J (2021). Dosimetry and optimal scan time of [(18)F]SiTATE-PET/CT in patients with neuroendocrine tumours. Eur J Nucl Med Mol Imaging.

[17] Ilhan H, Lindner S, Todica A, Cyran CC, Tiling R, Auernhammer CJ, Spitzweg C, Boeck S, Unterrainer M, Gildehaus FJ (2020). Biodistribution and first clinical results of (18)F-SiFAlin-TATE PET: a novel (18)F-labeled somatostatin analog for imaging of neuroendocrine tumors. Eur J Nucl Med Mol Imaging.

[18] Shah MH, Goldner WS, Halfdanarson TR, Bergsland E, Berlin JD, Halperin D, Chan J, Kulke MH, Benson AB, Blaszkowsky LS (2018). NCCN Guidelines Insights: neuroendocrine and adrenal tumors, Version 2.2018. J Natl Compr Cancer Network: JNCCN.

[19] Bozkurt MF, Virgolini I, Balogova S, Beheshti M, Rubello D, Decristoforo C, Ambrosini V, Kjaer A, Delgado-Bolton R, Kunikowska J (2017). Guideline for PET/CT imaging of neuroendocrine neoplasms with (68)Ga-DOTA-conjugated somatostatin receptor targeting peptides and (18)F-DOPA. Eur J Nucl Med Mol Imaging.

[20] Ambrosini V, Kunikowska J, Baudin E, Bodei L, Bouvier C, Capdevila J, Cremonesi M, de Herder WW, Dromain C, Falconi M (2021). Consensus on molecular imaging and theranostics in neuroendocrine neoplasms. Eur J cancer (Oxford England: 1990).

[21] Pavel M, Öberg K, Falconi M, Krenning EP, Sundin A, Perren A, Berruti A (2020). Gastroenteropancreatic neuroendocrine neoplasms: ESMO Clinical Practice Guidelines for diagnosis, treatment and follow-up. Annals of oncology: official journal of the European Society for Medical Oncology.

[22] Baudin E, Caplin M, Garcia-Carbonero R, Fazio N, Ferolla P, Filosso PL, Frilling A, de Herder WW, Hörsch D, Knigge U (2021). Lung and thymic carcinoids: ESMO Clinical Practice Guidelines for diagnosis, treatment and follow-up(☆). Annals of oncology: official journal of the European Society for Medical Oncology.

[23] Rinke A, Wiedenmann B, Auernhammer C, Bartenstein P, Bartsch DK, Begum N, Faiss S, Fottner C, Gebauer B, Goretzki P (2018). Practice guideline neuroendocrine tumors - AWMF-Reg. Z Gastroenterol.

[24] Zidan L, Iravani A, Kong G, Akhurst T, Michael M, Hicks RJ (2021). Theranostic implications of molecular imaging phenotype of well-differentiated pulmonary carcinoid based on (68)Ga-DOTATATE PET/CT and (18)F-FDG PET/CT. Eur J Nucl Med Mol Imaging.

[25] Moryoussef F, de Mestier L, Belkebir M, Deguelte-Lardière S, Brixi H, Kianmanesh R, Hoeffel C, Cadiot G (2017). Impact of liver and whole-body diffusion-weighted MRI for neuroendocrine tumors on Patient Management: a pilot study. Neuroendocrinology.

[26] d’Assignies G, Fina P, Bruno O, Vullierme MP, Tubach F, Paradis V, Sauvanet A, Ruszniewski P, Vilgrain V (2013). High sensitivity of diffusion-weighted MR imaging for the detection of liver metastases from neuroendocrine tumors: comparison with T2-weighted and dynamic gadolinium-enhanced MR imaging. Radiology.

[27] Frilling A, Li J, Malamutmann E, Schmid KW, Bockisch A, Broelsch CE (2009). Treatment of liver metastases from neuroendocrine tumours in relation to the extent of hepatic disease. Br J Surg.

[28] Panzuto F, Nasoni S, Falconi M, Corleto VD, Capurso G, Cassetta S, Di Fonzo M, Tornatore V, Milione M, Angeletti S (2005). Prognostic factors and survival in endocrine tumor patients: comparison between gastrointestinal and pancreatic localization. Endocrine-related Cancer.

[29] Madeira I, Terris B, Voss M, Denys A, Sauvanet A, Flejou JF, Vilgrain V, Belghiti J, Bernades P, Ruszniewski P (1998). Prognostic factors in patients with endocrine tumours of the duodenopancreatic area. Gut.

[30] Jackson T, Darwish M, Cho E, Nagatomo K, Osman H, Jeyarajah DR (2021). 68Ga-DOTATATE PET/CT compared to standard imaging in metastatic neuroendocrine tumors: a more sensitive test to detect liver metastasis?. Abdom Radiol (New York).

[31] Armbruster M, Zech CJ, Sourbron S, Ceelen F, Auernhammer CJ, Rist C, Haug A, Singnurkar A, Reiser MF, Sommer WH (2014). Diagnostic accuracy of dynamic gadoxetic-acid-enhanced MRI and PET/CT compared in patients with liver metastases from neuroendocrine neoplasms. J Magn Reson imaging: JMRI.

[32] Sadowski SM, Neychev V, Millo C, Shih J, Nilubol N, Herscovitch P, Pacak K, Marx SJ, Kebebew E (2016). Prospective study of 68Ga-DOTATATE Positron Emission Tomography/Computed tomography for detecting gastro-entero-pancreatic neuroendocrine tumors and unknown primary Sites. J Clin oncology: official J Am Soc Clin Oncol.

[33] Frilling A, Sotiropoulos GC, Radtke A, Malago M, Bockisch A, Kuehl H, Li J, Broelsch CE (2010). The impact of 68Ga-DOTATOC positron emission tomography/computed tomography on the multimodal management of patients with neuroendocrine tumors. Ann Surg.

[34] Hofman MS, Kong G, Neels OC, Eu P, Hong E, Hicks RJ (2012). High management impact of Ga-68 DOTATATE (GaTate) PET/CT for imaging neuroendocrine and other somatostatin expressing tumours. J Med Imaging Radiat Oncol.

[35] Barrio M, Czernin J, Fanti S, Ambrosini V, Binse I, Du L, Eiber M, Herrmann K, Fendler WP (2017). The impact of somatostatin Receptor-Directed PET/CT on the management of patients with neuroendocrine tumor: a systematic review and Meta-analysis. J nuclear medicine: official publication Soc Nuclear Med.

[36] Froelich MF, Schnitzer ML, Holzgreve A, Gassert FG, Gresser E, Overhoff D, Schwarze V, Fabritius MP, Nörenberg D, von Münchhausen N (2021). Cost-effectiveness analysis of 68Ga DOTA-TATE PET/CT, 111In-Pentetreotide SPECT/CT and CT for diagnostic workup of neuroendocrine tumors. Diagnostics.

[37] Schreiter NF, Brenner W, Nogami M, Buchert R, Huppertz A, Pape UF, Prasad V, Hamm B, Maurer MH (2012). Cost comparison of 111In-DTPA-octreotide scintigraphy and 68Ga-DOTATOC PET/CT for staging enteropancreatic neuroendocrine tumours. Eur J Nucl Med Mol Imaging.

[38] Van Essen M, Krenning EP, De Jong M, Valkema R, Kwekkeboom DJ (2007). Peptide receptor Radionuclide Therapy with radiolabelled somatostatin analogues in patients with somatostatin receptor positive tumours. Acta Oncol (Stockholm Sweden).

[39] Strosberg J, Wolin E, Chasen B, Kulke M, Bushnell D, Caplin M, Baum RP, Kunz P, Hobday T, Hendifar A (2018). Health-Related quality of life in patients with Progressive Midgut neuroendocrine tumors treated with (177)Lu-Dotatate in the phase III NETTER-1 trial. J Clin oncology: official J Am Soc Clin Oncol.

[40] Strosberg J, El-Haddad G, Wolin E, Hendifar A, Yao J, Chasen B, Mittra E, Kunz PL, Kulke MH, Jacene H (2017). Phase 3 trial of (177)Lu-Dotatate for Midgut neuroendocrine tumors. N Engl J Med.

[41] Sarmiento JM, Heywood G, Rubin J, Ilstrup DM, Nagorney DM, Que FG (2003). Surgical treatment of neuroendocrine metastases to the liver: a plea for resection to increase survival. J Am Coll Surg.

[42] Hoffmann RT, Paprottka PM, Schon A, Bamberg F, Haug A, Durr EM, Rauch B, Trumm CT, Jakobs TF, Helmberger TK (2012). Transarterial hepatic yttrium-90 radioembolization in patients with unresectable intrahepatic cholangiocarcinoma: factors associated with prolonged survival. Cardiovasc Interv Radiol.

[43] Bauckneht M, Albano D, Annunziata S, Santo G, Guglielmo P, Frantellizzi V, Branca A, Ferrari C, Vento A, Mirabile A (2020). Somatostatin receptor PET/CT imaging for the detection and staging of pancreatic NET: a systematic review and Meta-analysis. Diagnostics.

[44] Schroeder PR, Haugen BR, Pacini F, Reiners C, Schlumberger M, Sherman SI, Cooper DS, Schuff KG, Braverman LE, Skarulis MC (2006). A comparison of short-term changes in health-related quality of life in thyroid carcinoma patients undergoing diagnostic evaluation with recombinant human thyrotropin compared with thyroid hormone withdrawal. J Clin Endocrinol Metab.

[45] Holzapfel K, Eiber MJ, Fingerle AA, Bruegel M, Rummeny EJ, Gaa J (2012). Detection, classification, and characterization of focal liver lesions: value of diffusion-weighted MR imaging, gadoxetic acid-enhanced MR imaging and the combination of both methods. Abdom Imaging.

[46] Ruf J, Schiefer J, Furth C, Kosiek O, Kropf S, Heuck F, Denecke T, Pavel M, Pascher A, Wiedenmann B (2011). 68Ga-DOTATOC PET/CT of neuroendocrine tumors: spotlight on the CT phases of a triple-phase protocol. J nuclear medicine: official publication Soc Nuclear Med.

[47] Gabriel M, Decristoforo C, Kendler D, Dobrozemsky G, Heute D, Uprimny C, Kovacs P, Von Guggenberg E, Bale R, Virgolini IJ (2007). 68Ga-DOTA-Tyr3-octreotide PET in neuroendocrine tumors: comparison with somatostatin receptor scintigraphy and CT. J nuclear medicine: official publication Soc Nuclear Med.

[48] Haug AR, Cindea-Drimus R, Auernhammer CJ, Reincke M, Wängler B, Uebleis C, Schmidt GP, Göke B, Bartenstein P, Hacker M (2012). The role of 68Ga-DOTATATE PET/CT in suspected neuroendocrine tumors. J nuclear medicine: official publication Soc Nuclear Med.

[49] Sanli Y, Garg I, Kandathil A, Kendi T, Zanetti MJB, Kuyumcu S, Subramaniam RM (2018). Neuroendocrine tumor diagnosis and management: (68)Ga-DOTATATE PET/CT. AJR Am J Roentgenol.

[50] Yu J, Cao F, Zhao X, Xie Q, Lu M, Li J, Yang Z, Sun Y (2022). Correlation and comparison of somatostatin receptor type 2 immunohistochemical Scoring Systems with 68Ga-DOTATATE Positron Emission Tomography/Computed Tomography Imaging in Gastroenteropancreatic Neuroendocrine Neoplasms. Neuroendocrinology.

[51] Imperiale A, Boursier C, Sahakian N, Ouvrard E, Chevalier E, Sebag F, Addeo P, Taïeb D (2022). Value of (68)Ga-DOTATOC and Carbidopa-Assisted (18)F-DOPA PET/CT for Insulinoma localization. J nuclear medicine: official publication Soc Nuclear Med.

[52] Shah R, Sehemby M, Garg R, Purandare N, Hira P, Mahajan A, Lele V, Malhotra G, Verma P, Rojekar A (2022). 68) Ga-DOTATATE PET/CT imaging in endogenous hyperinsulinemic hypoglycemia: a tertiary endocrine centre experience. Clin Endocrinol (Oxf).

[53] Poeppel TD, Binse I, Petersenn S, Lahner H, Schott M, Antoch G, Brandau W, Bockisch A, Boy C (2011). 68Ga-DOTATOC versus 68Ga-DOTATATE PET/CT in functional imaging of neuroendocrine tumors. J nuclear medicine: official publication Soc Nuclear Med.

[54] Kabasakal L, Demirci E, Ocak M, Decristoforo C, Araman A, Ozsoy Y, Uslu I, Kanmaz B (2012). Comparison of ^68^Ga-DOTATATE and ^68^Ga-DOTANOC PET/CT imaging in the same patient group with neuroendocrine tumours. Eur J Nucl Med Mol Imaging.

[55] Velikyan I, Sundin A, Sörensen J, Lubberink M, Sandström M, Garske-Román U, Lundqvist H, Granberg D, Eriksson B (2014). Quantitative and qualitative intrapatient comparison of 68Ga-DOTATOC and 68Ga-DOTATATE: net uptake rate for accurate quantification. J nuclear medicine: official publication Soc Nuclear Med.

[56] Sandström M, Velikyan I, Garske-Román U, Sörensen J, Eriksson B, Granberg D, Lundqvist H, Sundin A, Lubberink M (2013). Comparative biodistribution and radiation dosimetry of 68Ga-DOTATOC and 68Ga-DOTATATE in patients with neuroendocrine tumors. J nuclear medicine: official publication Soc Nuclear Med.

[57] Choi SJ, Choi SH, Lee DY, Lee JS, Kim DW, Jang JK. Diagnostic value of [(68) Ga]Ga-DOTA-labeled-somatostatin analogue PET/MRI for detecting liver metastasis in patients with neuroendocrine tumors: a systematic review and meta-analysis. *European radiology* 2022. 10.1007/s00330-021-08527-z 35092473

