# Supplementary material for: Diagnostic performance of PET/CT in the detection of liver metastases in well-differentiated NETs
Source: Cancer Imaging. 2023 Apr 25;23:41. doi: 10.1186/s40644-023-00556-9 (PMC10131442; doi:10.1186/s40644-023-00556-9)
Supplement: Supplementary file 1 — Supplementary Material 1 [file 40644_2023_556_MOESM1_ESM.docx]

**Manuscript ID: CAIG-D-23-00022
Manuscript Title: Diagnostic performance of PET/CT in the detection of liver metastases in well-differentiated NETs**

Dear Dr. Beauregard,

Thank you very much for the opportunity to revise our manuscript for a resubmission. We appreciate the very helpful and constructive comments by the reviewers. We are convinced that our revisions, addressing each of the reviewers’ points in detail, have led to an improvement of our manuscript. In the following pages, we will respond to each section of the reviewers’ comments. We are confident that the manuscript now meets the quality standards of *Cancer Imaging* for publication. If you have any further questions, please do not hesitate to contact us.

With best regards,

Matthias P. Fabritius

**========================================**

**Reviewer reports**

**========================================**

**Reviewer #1:**

**Here is my review for your article entitled "Diagnostic performance of PET/CT in the detection of liver metastases in well-differentiated NETs". The results and discussion are clear and interesting. I only have the following minor comments to address:**

**Abstract**

**-	Page 2, line 31: Why put SSR between quotation marks?**

We entered SSR as the search term in our database and to underline this process we put SSR in quotation marks. We agree that this can be misleading and therefore deleted the quotation marks.

**-	Page 2, lines 37-38: You should pout at least one decimal after your % in parentheses. 16 false positives give 1%, and 51 false negatives give 2% when round up, which looks weird.**

We agree, thank you for this remark. We added one decimal to the numbers in the abstract and result section.

**-	Page 2, lines 41-42: Lesions < 1.2 cm are not small for PET imaging, at least for today's standard. I presume that it was indeed a source of error back in 2006. Did you observe an improvement in the false-negative rate between 2006 and 2021 as the scans improved in performance?**

Thank you very much for this very important remark and please excuse our misleading formulation of this important information.

Of the 36 mentioned “overlooked” small liver lesions only 3 lesions were 1.2 cm in size, one from 2017, one from 2012 and one from 2009. The majority of the remaining lesions were smaller than 0.6 cm (n=30), 3 lesions were 0.7 cm. However, the same PET/CT scanner was used in 2012 and 2017, so no overall improvement in PET scan performance, that definitely is present, can be inferred in this cohort.

To clarify this, we rewrote the results:

Abstract:

*“The most frequent reason for false-negative results was the small size of lesions with the majority <0.6 cm.”*

Results, line 172 ff:

*“The most frequent reason for the lack of SSR-uptake in the SSR-PET/CT was the small size of lesions in patients with overall low hepatic involvement (n=36), with mostly lesions < 0.6 cm (n=30), and very few lesions with 0.7 cm (n=3) and 1.2 cm (n=3).”*

**-	Related to the last comment, MRI also improved through the 15-year interval of your retrospective study. Do the false-positive rate also vary from the beginning to the end of the timeline?**

This is also an important remark. We reviewed the false-positive rated scans (n=16) and found that the distribution was very homogeneous over the years as followed:

| Year of scan | Number of patients |
| --- | --- |
| 2008 | 1 |
| 2009 | 1 |
| 2010 | 2 |
| 2012 | 3 |
| 2014 | 1 |
| 2015 | 1 |
| 2016 | 1 |
| 2017 | 1 |
| 2018 | 2 |
| 2019 | 1 |
| 2020 | 2 |

**Methods**

**-	Page 4, line 94: On a total of 8077 total patients, 1000 were investigated. Did you arbitrarily choose this number of patients, or your inclusion/exclusion criteria selected for exactly 1000 patients? The chart in Figure 1 seems to confirm that it is indeed inclusion/exclusion that selected for this number, however you should add more details in the Methods on how you selected your patients for analysis**.

In fact, the number of exactly 1000 patients was coincidence and resulted as following:

The database search yielded 8077 consecutive SSR-PET/CT examinations; however, these were not sorted or summarized by individual patient. After exclusion of all duplicates and multiples a total of 2605 patients remained who had received at least one SSR-PET/CT. In addition to the NET patients relevant to the study this sample also included 642 meningioma patients who were not to be considered further. Since only patients who had a NET were to be used for this study all other NEN (paraganglioma, pheochromocytoma, etc.) were also excluded from the sample. We also excluded two patients who were minors at the time of the initial examination from the studied collective, as well as 326 non-NEN tumor patients or false findings (in case of the latter, errors occurred in the findings report, in which the term ‘SSR’ was erroneously used although it was not an examination with an SSR analogue).

Of the remaining 1570 patients, exactly 1000 had received at least one liver MRI in addition to at least one SSR-PET/CT examination. To ensure that the PET/CT and MRI examinations had appropriate comparability, only MRIs done within 3 months after the PET/CT were matched for analysis. This resulted in a total of 2383 temporally corresponding examinations, which were then compared with each other.

To clarify patient selection, we added following to the methods section, line 94 ff:

*“These imaging studies were assigned to a total of 2605 patients. All studies listed multiple times to one patient, studies of patients <18 years or of other entities than G1/G2 NETs of the aerodigestive tract (e.g., meningioma, paraganglioma) were excluded. Of the therefore remaining 1570 patients, exactly 1000 patients received at least one MRI of the liver within 3 months after a matching SSR-PET/CT study.*

**-	Page 4, line 96: "multiparametric liver MRI imaging in an interval of max. +3 months". What do you mean? An interval of maximum 3 months, or an interval of +3 months? Please clarify.**

We apologize if this was misleading. Only MRI of the liver within 3 months after a matching SSR-PET/CT study were included. We rewrote the sentence.

-	**Page 5, line 105-107: SSR-PET was evaluated by 4 readers, but you only enumerate 3 (experienced physician, radiology resident, NM resident). At least you meant "experienced and board-certified radiologist and NM physicians". Please clarify.**

Indeed, this wording is unclear. Reading was performed by one experienced, board-certified radiologist as well as one board-certified nuclear medicine physician. Furthermore, one resident in radiology and one resident in nuclear medicine performed the reading. We adjusted the sentence accordingly.

Line 109 ff:

“*SSR-PET/CTs were evaluated by four readers: one experienced and board-certified radiologist, one experienced and board-certified nuclear medicine physician, one radiology resident and one nuclear medicine resident”*

**-	Page 5, line 111: "…and in unclear solitary lesions additionally pathological reports". Change "additionally" for "additional")**

We changed it accordingly.

**-	Page 5, line 116: Between 2006 and 2021 you used the same PET scanner? If any upgrade occurred in the meantime, you should mention it.**

Thank you for this important comment. Indeed, our PET/CT scanner changed in early 2008. Before 2008 the Discovery 64-slice PET/CT scanner (GE Healthcare) was used. Nevertheless, only 32 patients are included with imaging from 2007 and only one patient with imaging from 2006. None of these patients had false-positive or false-negative findings in our analysis. However, we apologize for our inaccuracy and added the old PET/CT scanner in our methods section.

**-	Page 6, line 130: Was there a difference in false-positive and false-negative rates according to when you used the 1.5 or the 3 T MRI as the true discovery benchmark?**

This is a very important point! In routine clinical practice at our centre, a 1.5 T MRI is commonly used for patients receiving liver MRI. Of all false-positive and false-negative imaging findings, only in 7 patients MRI was performed at a 3.0 T MRI. Thus no signal can be derived that in our cohort the 3.0 T device would be more accurate than the 1.5 T. For accuracy, all used MRI scanners are listed, but the routinely used MRI scanners were 1.5 T Magnetom Aera and Avanto. However, this is a very important information missing in the methods section so we adapted the paragraph to draw the reader's attention to this:

Line 134 ff:

*“Multiparametric MR examinations were mainly performed on a 1.5 T MR system (Magnetom Avanto, Magnetom Aera, Siemens Healthcare, Erlangen, Germany; Ingenia S, Philips Healthcare, Hamburg, Germany) and occasionally on a 3.0 T MR system (Verio, Skyra, Vida, Siemens Healthcare, Erlangen, Germany; Ingenia, Philips Healthcare, Hamburg, Germany) using a phased-array-coil for signal reception.”*

**Results**

**-	Page 7, line 157-160: For simplicity's sake, it would be better to describe only the % of confirmed hepatic involvement for MRI and PET without specifying the % of non-involvement, since we can easily deduct the latter knowing only the former…**

We deleted the non-hepatic involvement.

**-	Table 2 and Table 3: Table 2 seems to describe the false-positive cases (but is titled and referred as the false-negative table), whereas Table 3 details the false-negative cases (and is titled "false-positive). Please switch those tables.**

We apologize for this mistake. We have accidentally mixed up the position of the tables. The new version contains the correctly assigned tables.

**Discussion**

**-	Page 10, line 220: You should put "inter alia" in italics.**

We corrected the term accordingly.

**-	Page 10, line 225: …in more than every second patient. What do you mean, more than 50%? Please specify.**

Frilling et al. reported that in 31/52 patients (59.6%) 68Ga-DOTATOC-PET/CT altered treatment decision compared to CT and/or MRI alone. To prevent confusion, we rewrote the sentence as followed:

Line 236 ff:

*“Frilling et al. reported on the role of SSR-PET/CT in detection/staging of NET and altered treatment decisions in more than every second patient (31/52; 59.6%) compared to CT and/or MRI alone.”*

**-	Page 11, line 244-245: I should read: "In our retrospective study, analyzing patient-based 245 metastatic hepatic involvement, the most frequent reason…". The comma is misplaced in the sentence and should go after "involvement" instead of after "study".**

we corrected the sentence accordingly.

**-	Page 12, line 267: "The same was the case…" should be changed for "The same was observed…".**

We corrected the term accordingly.

**-	Page 12, line 273: Higher-grade instead of higher-grad.**

We corrected the term accordingly.

**Figure 1: The chart seems blurry. It might be because it is displayed full-page in the draft, but you should produce a higher-resolution image for publication in my opinion.**

Thank you, we agree with you. We will upload Figure 1 with a higher resolution for the final article.

**Reviewer #2:**

**The authors present a retrospective analysis of the performance of SSTR PET/CT for detection of hepatic metastases with reference standard MRI.**

**The paper is well written and presents retrospective data on a large number of patients, yielding important results for routine clinical practice.**

**A few minor comments:**

**1- While it is clear from the manuscript that the images of the MRI and SSTR PET-CT were reviewed side by side for the cases with the discrepant results, this is not clear from the abstract or methods. If this is indeed the case, this should be clarified.**

Thank you for this important remark. Indeed, in case of discrepant results SSR-PET/CT images and MRI was reviewed side by side by an independent reader to reach final consensus. To clarify this, we added following to the abstract and methods section:

Abstract:  *“In case of discrepant reports, MRI and PET/CT were reviewed side by side for consensus reading.”*

Methods, line 114 ff:

*”In case of divergent medical reports, additional imaging studies - and in unclear solitary lesions additional pathological reports - were reviewed side by side by an independent reader to find the underlying reason and reach final consensus.”*

**2- Is the difference of the percentage of false negative and false positive studies statistically significant?**

Thank you for this comment. We applied the chi-squared test to compare a possible statistically significant difference of the false-negative (n=51) and false-positive (n=16) imaging findings. Indeed, we found significantly more often false-negative than false-positive findings between the two groups (p=0.01).

The significantly fewer false-positive results emphasize the high specificity of SSR-PET/CT. In contrast to e.g. FDG-PET/CT which can accumulate in malignant or inflammatory lesions, SSR tracers show high specificity to somatostatin 2 and 5 receptors, which are overexpressed in NET (1). The higher false-negative rate is to be explained mainly due to the small lesion size which was under the detection rate of the PET-study.

To emphasize this point we added the analysis to the manuscript:

Methods, line 151 f*:*

*“Chi-squared test was used to compare the frequency of false-positive and false-negative imaging findings.”*

Results, line 200 f:

*“**False-negative findings were statistically significantly more frequent than false-positive findings (p < 0.01).”*

Discussion, line 285 ff:

*“False-negative findings were significantly more frequent than false-positive findings which emphasizes the high specificity of SSR-PET/CT. SSR tracers show high affinity to somatostatin 2 and 5 receptors, which are overexpressed in NET. The higher false-negative rate is to be explained mainly due to the above-mentioned small sub-centimeter lesion size which was under the detection rate of the PET-study. By the above-mentioned rate of small sub-centimeter lesions.”*

- *χ^2^ (2, N=2383) = 2077.387, p < 0.00001,* thus the difference is statistically significant.

References:

1. Haug AR, Cindea-Drimus R, Auernhammer CJ et al (2012) The role of 68Ga-DOTATATE PET/CT in suspected neuroendocrine tumors. J Nucl Med 53:1686-1692

**3- False positives are defined as SSTR uptake with MRI lesion. However, in table 3 listing the false positive studies, many of the findings in SSR-PET are listed as no SSR uptake- these should therefore not be listed as false positives. If this does not represent a typo and is indeed true, then the statistics need to updated.**

Thank you very much for bringing this error to our attention. We indeed mixed up the tables. The new uploaded version of the manuscript contains the correctly assigned tables 2 and 3.

**Additionally, in the figure provided for false positives, how did you ensure that this focus of SSTR uptake was not a true lesion adjacent to vascular structures. Was follow-up performed or other reference standards?**

Thank you very much for this important question. We performed multiple follow-up MRI and PET/CT scans and could therefore ensure that the lesion was not a true lesion. However, we thank you for drawing our attention to a mistake: the lesion is presumably more likely bile duct-associated and not vascular-associated. We corrected our mistake in the title of figure 3.
